# Circulating lncRNA ITSN1‐2 is upregulated, and its high expression correlates with increased disease severity, elevated inflammation, and poor survival in sepsis patients

**DOI:** 10.1002/jcla.22836

**Published:** 2019-02-25

**Authors:** Qingwei Zeng, Jingdong Wu, Shijiang Yang

**Affiliations:** ^1^ Department of Emergency Surgery The Central Hospital of Wuhan, Tongji Medical College, Huazhong University of Science and Technology Wuhan China

**Keywords:** disease risk, inflammatory cytokine, lncRNA ITSN1‐2, sepsis, survival

## Abstract

**Background:**

This study aimed to assess the correlation of long noncoding (lnc) RNA intersectin (ITSN) 1‐2 expression with disease risk, severity, inflammation, and survival in sepsis patients.

**Methods:**

Three hundred and nine intensive care unit (ICU)‐treated sepsis patients and 300 healthy controls were consecutively recruited in this study. Blood samples were collected from all sepsis patients within 24 hours after admitted to ICU and from healthy controls at the time of health screening, and the expression of lncRNA ITSN1‐2 in plasma was detected by quantitative polymerase chain reaction. Disease severity was assessed by physicians using acute physiology and chronic health evaluation (APACHE) II score on day 1 after ICU admission. Additionally, the plasma inflammatory cytokines (including tumor necrosis factor α (TNF‐α), interleukin 1β (IL‐1β), IL‐6, IL‐8, IL‐10, and IL‐17) were measured by enzyme‐linked immunosorbent assay (ELISA) kits.

**Results:**

lncRNA ITSN1‐2 was highly expressed in sepsis patients compared to healthy controls and could differentiate sepsis patients from healthy controls with area under the curve (AUC) 0.777 (95% CI: 0.740‐0.813). lncRNA ITSN1‐2 expression was positively correlated with APACHE II score, C‐reactive protein (CRP), TNF‐α, IL‐6, and IL‐8 levels, but negatively correlated with IL‐10 level. In addition, lncRNA ITSN1‐2 was highly expressed in non‐survivors compared to survivors and could distinguish survivors from non‐survivors in sepsis patients with AUC 0.654 (95% CI: 0.581‐0.726).

**Conclusion:**

Circulating lncRNA ITSN1‐2 is upregulated, and its high expression associates with increased disease severity and inflammation as well as poor prognosis in sepsis patients.

## 1 INTRODUCTION

Sepsis, characterized as severe systemic inflammation, is defined as life‐threatening organ dysfunction caused by a dysregulated host response to infection, which is a global disease burden with rising incidence and severity, especially in developing countries.^1^, ^2^ As in China, the incidence of sepsis is up to 37.3% and the mortality of sepsis ranges from 33.5% to 48.7% in intensive care unit (ICU) setting patients, and its mortality is approximately onefold higher than the rates among developed countries.^3^, ^4^, ^5^, ^6^ Although more and more diagnostic markers have been discovered and analytical techniques have improved, the delay of diagnosis for sepsis is still very common due to acute, critical illness, contributing to poor treatment outcomes, increased hospitalization duration and cost, and even death. Therefore, exploring reliable sepsis biomarker for early diagnosis and prognostic prediction is an urgent need for helping establish clinical treatment strategies.

Long noncoding (lnc) RNA is class of RNA longer than 200 nucleotides and does not encode proteins.^7^ Accumulating studies have revealed that multiple lncRNAs are implicated in acute inflammatory response against microbial infection through transcriptionally regulating inflammatory gene expression.^7^ lncRNA intersectin (ITSN) 1‐2 is one of lncRNA family members located in chromosome 21 with its NONCODE gene ID NONHSAG032726.2, and its length was 451 bp with NONCODE transcript ID NONHSAT081856.2, starting from 33976355 to end site 33976982.^8^ It is reported that lncRNA ITSN1‐2 high expression is correlated with increased disease risk and activity as well as inflammation degree of rheumatoid arthritis (RA).^8^ Considering that RA and sepsis are both characterized as systematic inflammatory response and the latter presents with even more severe inflammation, and our preliminary research in a small population observed that lncRNA ITSN1‐2 was extremely upregulated in sepsis patients, we speculated that lncRNA ITSN1‐2 may be involved in the development and progression of sepsis. Thus, the objective of this study was to assess whether lncRNA ITSN1‐2 could distinguish sepsis from healthy controls, and its correlation with disease severity, inflammation, and survival in sepsis patients.

## 2 MATERIALS AND METHODS

### 2.1 Participants

Between January 2015 and December 2017, 309 sepsis patients who were admitted to ICU of The Central Hospital of Wuhan, Tongji Medical College, Huazhong University of Science and Technology, and 300 healthy controls who underwent physical examinations at Health Screening Centre were consecutively recruited in this study, and the controls were recruited in the same period of sepsis patients. For the sepsis patients, the inclusion criteria were as follows: (1) diagnosed as sepsis according to the American College of Chest Physicians and the Society of Critical Care Medicine criteria (ACCP/SCCM)^9^; (2) age above 18 years old. And the exclusion criteria were as follows: (1) patients who continuously used glucocorticoids and immunosuppressive drugs within 6 months; (2) patients who previously underwent chemotherapy, radiotherapy, immunotherapy, or endocrinotherapy for other diseases; (3) patients with acquired immune deficiency syndrome (AIDS); (4) patients who were suffering from malignancies; (5) patients who died within 24 hours after admitted in ICU; and (6) patients who were pregnant. As for the healthy controls, the exclusion criteria were as follows: age <18 years old, had any symptoms of active infection or any history of immune disease, inflammatory disease, severe infection, cancer, hematologic malignancies, pregnancy, or on current chronic steroid therapy. This study was approved by the Ethics Committee of The Central Hospital of Wuhan, Tongji Medical College, Huazhong University of Science and Technology, and written informed consents were obtained from all participants or their authorized representatives.

### 2.2 Samples and data collection

Blood samples were collected from all sepsis patients within 24 hours after admitted to ICU and from healthy controls at the time of health screening. Also, the characteristics of sepsis patients were recorded including age, gender, and BMI, and the levels of serum creatinine (Scr), albumin, white blood cell (WBC), and C‐reactive protein (CRP) were assayed routinely. In addition, disease severity of sepsis was assessed by physicians using acute physiology and chronic health evaluation (APACHE) II score on day 1 after ICU admission. All patients were treated in accordance with current guidelines for treatment of sepsis and followed up for 30 days (30‐day survival was recorded as well).

### 2.3 lncRNA ITSN1‐2 detection

Plasma was separated from blood samples by centrifugation, and the total RNA in plasma was extracted using the TRIzol (Invitrogen, USA) reagent following the manufacturer’s instructions. The complementary DNA (cDNA) synthesis with extracted total RNA was performed using a PrimeScript™ RT Reagent Kit (Takara, China). All quantitative RNA detections were carried out in triplicates on an Applied Biosystems 7900 HT Thermocycler (Applied Biosystems, USA) with TB Green™ Fast qPCR Mix (Takara, China). The reaction was incubated for at 95°C for 3 minutes, followed by 40 cycles of 95°C for 5 seconds and 61°C for 30 seconds. Relative quantitation of gene expression was analyzed by the 2^−ΔΔCt^ method, and the reduced glyceraldehyde‐phosphate dehydrogenase (GAPDH) was utilized as an internal control for normalization of lncRNA expression. The primers were as follows: lncRNA ITSN1‐2 forward: TTAGTCTGTTCAGGCTGCCATAA, reverse: GCTTGCTCACTTGCTATCTCTTG; GAPDH forward: GAGTCCACTGGCGTCTTCAC, reverse: ATCTTGAGGCTGTTGTCATACTTCT.

### 2.4 Detections of inflammatory cytokines for sepsis patients

The plasma inflammatory cytokines of sepsis patients were measured by enzyme‐linked immunosorbent assay (ELISA) kits (Beijing Wantai Biological Pharmaceutical Co., Ltd., China) according to the protocols provided by the manufacturer, which included tumor necrosis factor α (TNF‐α), interleukin 1β (IL‐1β), IL‐6, IL‐8, IL‐10, and IL‐17.

### 2.5 Statistical analysis

Statistical analysis was performed using SPSS 22.0 software (SPSS Inc, USA) and GraphPad Prism 6.01 (GraphPad Software, USA). Normal distributed continuous variable was presented as mean value ± standard deviation, skewed distributed continuous variable was expressed as median (25th‐75th quantiles), and categorized variable was presented as count (percentage). Differences of lncRNA ITSN1‐2 relative expression were determined by Wilcoxon rank‐sum test. Correlation analyses were performed using Spearman’s test. Receiver operating characteristic (ROC) curves and the area under the ROC curve were used to assess the diagnostic value of lncRNA ITSN1‐2 levels for sepsis and the ability to discriminate between survivors and non‐survivors. All tests were all two‐sided, and *P*‐value <0.05 was considered significant.

## 3 RESULTS

### 3.1 Baseline characteristics

There were 309 patients in sepsis group, including 204 (66.0%) males as well as 105 (34.0%) females, and their mean age was 57.3 ± 9.7 years (Table 1). The median APACHE II score was 16.0 (12.0‐20.0), and the median CRP level was 48.31 (29.58‐83.80) mg/L. In addition, the median levels of TNF‐α, IL‐1β, IL‐6, IL‐8, IL‐10, and IL‐17 were 60.34 (35.50‐95.63), 4.53 (2.53‐8.43), 71.35 (42.89‐115.91), 68.49 (36.64‐115.76), 26.95 (16.26‐52.54), and 48.77 (23.86‐77.11) pg/Ml, respectively. Other detailed information of baseline characteristics in sepsis patients is shown in Table 1. As for healthy controls, there were 300 cases, including 185 (61.7%) males as well as 115 (38.3%) females, and their mean age was 55.8 ± 9.7 years. No difference of demographic characteristics between sepsis group and healthy controls was found (All *P* > 0.05).

**Table 1:** Baseline characteristics of patients with sepsis

Characteristics	Sepsis patients (N = 309)
Age (years)	57.3 ± 9.7
Gender (n/%)	
Male	204 (66.0)
Female	105 (34.0)
BMI (kg/m^2^)	23.1 ± 4.6
Scr (mg/dL)	1.51 (1.07‐2.09)
Albumin (g/L)	26.83 (21.30‐35.30)
WBC (10^9^/L)	13.81 (3.92‐28.06)
CRP (mg/L)	48.31 (29.58‐83.80)
APACHE II score	16.0 (12.0‐20.0)
TNF‐α (pg/mL)	60.34 (35.50‐95.63)
IL‐1β (pg/mL)	4.53 (2.53‐8.43)
IL‐6 (pg/mL)	71.35 (42.89‐115.91)
IL‐8 (pg/mL)	68.49 (36.64‐115.76)
IL‐10 (pg/mL)	26.95 (16.26‐52.54)
IL‐17 (pg/mL)	48.77 (23.86‐77.11)

Data were presented as mean value ± standard deviation, count (percentage), or median (25th‐75th quantiles). BMI, body mass index; Scr, serum creatinine; WBC, white blood cell; CRP, C‐reactive protein; APACHE, acute physiology and chronic health evaluation; TNF, tumor necrosis factor; IL, interleukin.

### 3.2 lncRNA ITSN1‐2 expression in sepsis patients and healthy controls

**Figure 1:**
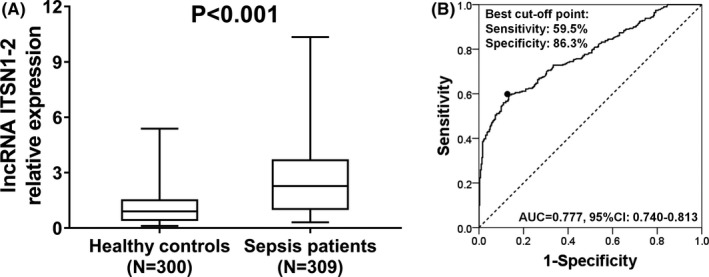
Comparison of lncRNA ITSN1‐2 expression between sepsis patients and healthy controls. A, LncRNA ITSN1‐2 expression was higher in sepsis patients compared to healthy controls (median value 2.271 vs 0.903). B, ROC curve displayed that lncRNA ITSN1‐2 present good diagnostic value of sepsis, and its expression was 1.820 at best cutoff point that was defined as the point where the sum of sensitivity and specificity was maximum. Wilcoxon rank‐sum test was performed to compare lncRNA ITSN1‐2 expression between sepsis patients and healthy controls. LncRNA ITSN1‐2, long noncoding RNA intersectin 1‐2; ROC, receiver operating characteristic

The lncRNA ITSN1‐2 expression was higher in sepsis patients compared to healthy controls (*P* < 0.001) (Figure 1A). The ROC curve disclosed that lncRNA ITSN1‐2 expression could distinguish sepsis patients from healthy controls with area under the curve (AUC) 0.777 (95% confidence interval (CI): 0.740‐0.813) (Figure 1B). The sensitivity and specificity were 59.5% and 86.3% at the best cutoff point that was defined as the point where the sum of sensitivity and specificity was maximum, and lncRNA ITSN1‐2 expression was 1.820. These data suggested that lncRNA ITSN1‐2 might be a biomarker for sepsis risk.

### 3.3 Correlation of lncRNA ITSN1‐2 expression with APACHE II score, CRP level, and inflammatory cytokines

**Figure 2:**
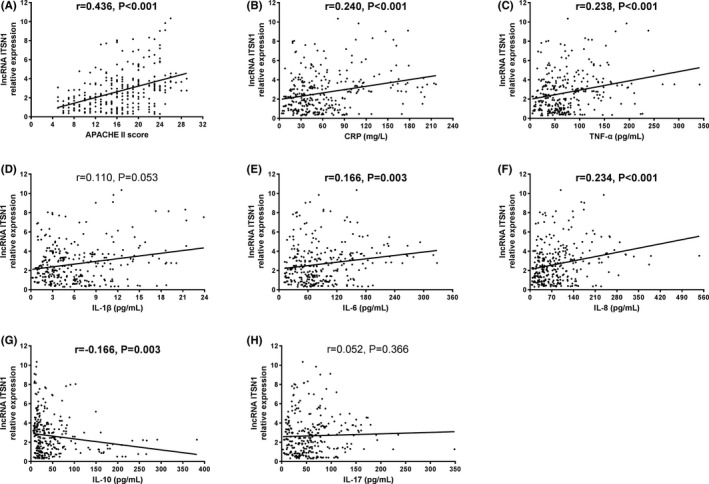
Correlation of lncRNA ITSN1‐2 expression with APACHE II score. A, LncRNA ITSN1‐2 expression was positively associated with APACHE II score in sepsis patients. B, LncRNA ITSN1‐2 expression was positively associated with CRP level in sepsis patients. LncRNA ITSN1‐2 expression was positively correlated with TNF‐α (C), IL‐6 (E), and IL‐8 levels (F), while negatively correlated with IL‐10 level (G) and there was no correlation of lncRNA ITSN1‐2 expression with IL‐1β (D) and IL‐17 levels (H). Spearman’s test was performed for correlation analyses. lncRNA ITSN1‐2, long noncoding RNA intersectin 1‐2; APACHE, acute physiology and chronic health evaluation. CRP, C‐reactive protein. TNF‐α, tumor necrosis factor α; IL, interleukin

lncRNA ITSN1‐2 expression was positively correlated with APACHE II score (*r* = 0.436, *P* < 0.001) (Figure 2A). In addition, higher lncRNA ITSN1‐2 expression was associated with increased CRP level (*r* = 0.240, *P* < 0.001) (Figure 2B). As to inflammatory cytokines, lncRNA ITSN1‐2 expression was positively correlated with TNF‐α (*r* = 0.238, *P* < 0.001) (Figure 2C), IL‐6 (*r* = 0.166, *P* = 0.003) (Figure 2E), and IL‐8 levels (*r* = 0.234, *P* < 0.001) (Figure 2F), but negatively correlated with IL‐10 level (*r* = −0.166, *P* = 0.003) (Figure 2G), while no correlation of lncRNA ITSN1‐2 expression with IL‐1β (*P* = 0.053) (Figure 2D) and IL‐17 levels (*P* = 0.366) (Figure 2H) was observed. These data indicated that lncRNA ITSN1‐2 might be a biomarker for disease severity and inflammation in sepsis patients.

### 3.4 Comparison of lncRNA ITSN1‐2 expression between non‐survivors and survivors

**Figure 3:**
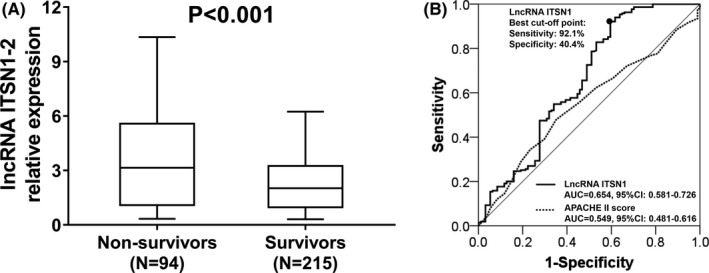
Comparison of lncRNA ITSN1‐2 expression between sepsis non‐survivors and survivors. A, LncRNA ITSN1‐2 expression was higher in sepsis non‐survivors compared to survivors (median value 3.146 vs 2.018). B, ROC curve displayed that lncRNA ITSN1‐2 present good diagnostic value of survival, and its expression was 4.059 at best cutoff point that was defined as the point where the sum of sensitivity and specificity was maximum. Wilcoxon rank‐sum test was performed to compare lncRNA ITSN1‐2 expression between sepsis non‐survivors and survivors. LncRNA ITSN1‐2, long noncoding RNA intersectin 1‐2; ROC, receiver operating characteristic

The lncRNA ITSN1‐2 expression was decreased in survivors compared to non‐survivors (*P* < 0.001) (Figure 3A). The ROC curve revealed that the AUC of lncRNA ITSN1 and APACHE II score was 0.654 (95% CI: 0.581‐0.726) and 0.549 (95% CI: 0.481‐0.616), respectively, and lncRNA ITSN1‐2 expression present a good value on distinguishing survivors and non‐survivors in sepsis patients, whose sensitivity and specificity at the best cutoff point were 92.1% and 40.4%, respectively, and lncRNA ITSN1‐2 expression was 4.059, which implied that lncRNA ITSN1‐2 might be a prognostic marker in sepsis patients (Figure 3B). The detailed information about factors affecting mortality in sepsis patients by Cox regression analysis is shown in Supplementary Table 1.

## 4 DISCUSSION

In the present study, we observed that: (1) lncRNA ITSN1‐2 was highly expressed in sepsis patients compared to healthy controls, and it presented a good diagnostic value for sepsis; (2) lncRNA ITSN1‐2 expression was positively correlated with APACHE II score and inflammatory markers; and (3) lncRNA ITSN1‐2 was highly expressed in non‐survivors compared to survivors, and it could well distinguish survivors from non‐survivors in sepsis patients.

lncRNAs have been reported to be involved in multiple biological processes and present with various functions, including affecting the genes transcription, regulating epigenetic changes as well as managing protein activity via binding to proteins.^10^ Recently, several in vitro studies have disclosed that lncRNAs play critical roles in immune and inflammatory response.^11^, ^12^, ^13^, ^14^ For instance, lncRNA‐Cox2 increases IL‐6 and IL‐23 production following microbial infection via activating Toll‐like receptor (TLR) signaling pathway in both macrophages and dendritic cells (DC).^11^ Another inflammatory enhancer RNA (eRNA) called lncRNA IL‐eRNA promotes IL‐1β production by activating TLR signaling pathway in lipopolysaccharide (LPS)‐induced human monocytes.^12^ As to sepsis, several lncRNAs have been reported to act as positive or negative regulators.^13^, ^14^ For example, lncRNA HOX transcript antisense RNA (HOTAIR) facilitates TNF‐α production in cardiomyocytes of LPS‐induced sepsis mice by activating nuclear factor (NF)‐κB pathway.^13^ In addition, lncRNA interleukin‐7 receptor α‐subunit (IL‐7R) negatively regulates E‐selectin, vascular cell adhesion molecule‐1 (VCAM‐1), IL‐6, and IL‐8 production via interacting with the human IL‐7R gene, thereby attenuated the LPS‐induced proinflammatory response.^14^ Briefly, several lncRNAs could regulate inflammatory response in inflammatory diseases, particularly in sepsis.

In clinical trials, dysregulated lncRNAs are found in sepsis patients and these lncRNAs display good diagnostic values for sepsis.^15^, ^16^, ^17^ For instance, plasma lncRNA nuclear‐enriched abundant transcript (NEAT1) is highly expressed in sepsis patients compared to healthy controls, which could distinguish sepsis patients from healthy controls with AUC 0.730.^15^ Also, lncRNA ENST00000452391.1 is highly expressed in sepsis compared to healthy controls and ROC curve displays its good diagnostic value with AUC 0.866.^17^ However, for lncRNA ITSN1‐2, there is limited information about its role in inflammatory disease, just one study discloses that high expression of lncRNA ITSN1‐2 is observed in RA patients, and it presents with good diagnostic value (AUC 0.898). Given that sepsis arises from dysregulated inflammatory response to infection, even more severe than RA, we hypothesized that lncRNA ITSN1‐2 might have diagnostic value in sepsis. In the present study, we uncovered that the expression of circulating lncRNA ITSN1‐2 was elevated in sepsis patients compared to healthy controls and it presented good value on distinguishing sepsis patients from healthy controls with AUC 0.777, which might be caused by that lncRNA ITSN1‐2 was able to serve as a promoter in regulating expression of inflammation‐related proteins by activation of multiple inflammatory pathways, inducing systemic inflammatory response in sepsis patients; thereby, its expression was higher in sepsis patients. As to the usefulness of lncRNA ITSN1‐2 in disease severity and inflammation assessment, the clinical study about RA elucidates that lnc‐ITSN1‐2 is positively associated with erythrocyte sedimentation rate (ESR), CRP, and disease activity score in 28 joints (DAS28) in RA patients. In line with these previous studies, we discovered that high expression of lncRNA ITSN1‐2 was associated with increased APACHE II score, CRP, TNF‐α, IL‐6, and IL‐8 levels in sepsis patients. The possible explanation might be that lncRNA ITSN1‐2 perhaps facilitated inflammatory factors production via several inflammatory pathways (such as NF‐κB pathway and TLRs pathway), leading to aggravation of inflammation and exacerbation of organ dysfunction, thereby resulted in increased disease severity in sepsis patients.^18^, ^19^, ^20^ However, how lncRNA ITSN1‐2 modulates inflammatory responses and causes progression of disease severity in sepsis requires further studies on mechanism to validate it. In addition, based on several previous studies, many inflammatory cytokines (such as CRP) are positively correlated with sepsis risk and appear to be promising indicators for worse prognosis in sepsis patients.^21^, ^22^

Currently, the prognostic value of lncRNAs in sepsis has rarely been investigated and only a few lncRNAs are reported in this respect.^15^, ^17^ For example, lncRNA NEAT1 expression is higher in non‐survivors compared to survivors and present good predictive value for sepsis‐related survival with AUC 0.641.^15^ Moreover, lncRNA ENST00000452391.1 high expression is correlated with reduced survival in sepsis patients.^17^ Although above studies display the prognostic value of several lncRNAs in sepsis, there is no information about the role of lncRNA ITSN1‐2 in prognosis of sepsis patients. Thus, in this study, we discovered that lncRNA ITSN1‐2 was highly expressed in sepsis non‐survivors compared to survivors and ROC curve disclosed lncRNA ITSN1‐2 could distinguish survivors and non‐survivors with AUC 0.654 in sepsis patients, which could be explained by that: (1) lncRNA ITSN1‐2 contributed to elevated disease severity and systematic inflammation, which was validated in our aforementioned data, thereby caused poor prognosis in sepsis patients;^1^, ^3^ (2) lncRNA ITSN1‐2 might attenuate cell response to sepsis treatment, such as anti‐infection and anti‐inflammation drugs, thereby worsened prognosis in sepsis patients. However, this hypothesis needed further investigation to confirm.

Limitations existed in the present study. Firstly, all enrolled patients were from single center and ICU; thus, multicentric and population‐based sample was needed to validate the diagnostic and prognostic values of lncRNA ITSN1‐2 in sepsis. Secondly, the detailed mechanisms of how lncRNA ITSN1‐2 associated with inflammation and organ dysfunction were not explored. Thirdly, black race has been reported to be an important risk factor for the development of sepsis and a predictor of poor outcomes in sepsis compared to white race, while our study only investigated the role of lncRNA ITSN1‐2 in sepsis in the yellow race and whether the results were applicable to other races was unknown.^23^ Finally, we have performed the correlation of lncRNA ITSN1‐2 with inflammatory cytokines levels, and the poor correlations were observed (eg, with IL‐10 and IL‐17), which might be resulted from the extreme value that might be correlated with the regulation of lncRNA to influence inflammatory factors levels.

Summarily, circulating lncRNA ITSN1‐2 is upregulated and its high expression associates with increased disease severity and inflammation as well as poor prognosis in sepsis patients.

## CONFLICTS OF INTEREST

The authors declare that they have no conflicts of interest.
