# Supplementary material for: Circulating lncRNA ITSN1‐2 is upregulated, and its high expression correlates with increased disease severity, elevated inflammation, and poor survival in sepsis patients
Source: J Clin Lab Anal. 2019 Feb 25;33(4):e22836. doi: 10.1002/jcla.22836 (PMC6528565; doi:10.1002/jcla.22836)
Supplement: Supplementary file 1 [file JCLA-33-e22836-s001.docx]

**Supplementary Table 1.** Cox’s proportional hazards regression model analysis of factors affecting mortality

| Items | Univariate Cox’s regression | | | | Multivariate Cox’s regression | | | |
| --- | --- | --- | --- | --- | --- | --- | --- | --- |
|  | P value | HR | 95% CI | | P value | HR | 95% CI | |
|  |  |  | Lower | Higher |  |  | Lower | Higher |
| LncRNA ITSN1 relative expression | <0.001 | 1.320 | 1.215 | 1.433 | <0.001 | 1.419 | 1.274 | 1.580 |
| Age | 0.290 | 1.011 | 0.991 | 1.032 | 0.903 | 0.999 | 0.977 | 1.021 |
| Male | 0.447 | 0.850 | 0.560 | 1.292 | 0.316 | 0.796 | 0.509 | 1.244 |
| BMI | 0.076 | 0.959 | 0.916 | 1.004 | 0.242 | 0.972 | 0.927 | 1.019 |
| Scr | 0.475 | 1.057 | 0.908 | 1.229 | 0.304 | 0.890 | 0.713 | 1.111 |
| Albumin | 0.539 | 0.993 | 0.971 | 1.015 | 0.665 | 0.993 | 0.963 | 1.024 |
| WBC | 0.558 | 1.004 | 0.990 | 1.018 | 0.687 | 1.004 | 0.983 | 1.027 |
| CRP | 0.852 | 1.000 | 0.996 | 1.005 | 0.386 | 0.995 | 0.985 | 1.006 |
| APACHE II score | 0.182 | 1.024 | 0.989 | 1.060 | 0.159 | 0.968 | 0.926 | 1.013 |
| TNF-α | 0.874 | 1.000 | 0.997 | 1.004 | 0.158 | 0.992 | 0.981 | 1.003 |
| IL-1β | 0.430 | 1.016 | 0.976 | 1.059 | 0.210 | 1.038 | 0.979 | 1.100 |
| IL-6 | 0.843 | 1.000 | 0.997 | 1.004 | 0.960 | 1.000 | 0.994 | 1.006 |
| IL-8 | 0.711 | 1.000 | 0.998 | 1.003 | 0.423 | 1.002 | 0.997 | 1.008 |
| IL-10 | 0.276 | 0.998 | 0.993 | 1.002 | 0.112 | 0.995 | 0.989 | 1.001 |
| IL-17 | 0.939 | 1.000 | 0.995 | 1.004 | 0.825 | 0.999 | 0.994 | 1.005 |

Factors affecting mortality were determined by univariate and multivariate Cox’s proportional hazards regression model analyses. P value < 0.05 was considered significant.

BMI: body mass index; Scr: serum creatinine; WBC: white blood cell; CRP: C-reactive protein; APACHE: acute physiology and chronic health evaluation; TNF-α: tumor necrosis factor-α; IL: interleukin.
